# Advertisements in Voluntary Hospitals

**Published:** 1916-07-01

**Authors:** Chas. F. Simond

**Affiliations:** Manager.


*To the Editor of* THE HOSPITAL.

### Advertisements in Voluntary Hospitals.

*To the Editor of* THE HOSPITAL.

SIR,—Our attention has been called to your article on "Advertisements in Voluntary Hospitals" in your issue of June 24. Why will not newspaper writers ascertain the facts before they make their criticisms? Had you taken this trouble you would have found that the hospital in question appealed to the public for funds and sent an appeal to us, offering to name a cot in return for a donation of £400; and that we accepted the offer on condition that they would appoint a competent person to inquire fully into any fifty of the cases reported upon in our "Baby Book," 1915.

You will see, therefore, that you have entirely misrepresented the matter, to our disadvantage, making it appear that there was no generosity at all about our offer, but that our object was merely the sordid one of obtaining an advertisement in return for payment.

If it is wrong for hospitals "to accept advertisements which are tantamount to a perpetual advertisement," why do they make such offers? Would not a Pears' Cot be as much a perpetual advertisement of Pears' Soap as a Nestlé Cot would of Nestlé's Milk? And what if Dr. Nestlé, the famous editor of the Greek Testament, or Mr. Nestlé, the well-known hair-dresser, should found a Nestlé Cot? Would it not perpetuate the fame of Nestlé's Milk just as much as if we were the donors of the endowment?

To prove that you have taken an erroneous view of the case, and that our main object was to get the effect of Nestlé's Milk on infants investigated, not to "turn the walls of a hospital into an advertisement hoarding," we need only say that, in response to another appeal, we offered the Great Ormond Street Hospital for Sick Children (the other hospital having declined our offer) £250 on the sole condition that they "appoint a competent person to inquire fully into any fifty of the cases reported in our 'Baby Book' for 1915 and 1916." There was no question here of naming a cot or getting an advertisement of any kind, permanent or otherwise.

If you ask why we are anxious for hospitals to investigate the effect of feeding infants on Nestlé's Milk, we reply that while the reports received from parents in response to our own investigations convince us of its advantages, we find it difficult to get such evidence scrutinised by medical men, many of whom seem to attach more weight to theories found in books than to actual facts. Trusting that you will insert this letter in your next issue, we are, Sir, yours faithfully,

NESTLÉ AND ANGLO-SWISS CONDENSED MILK CO.,
London, Home Department.

June 27, 1916. CHAS. F. SIMOND, Manager.

[We consider that the hospital which approached Messrs. Nestlé with an offer to name a cot in return for a donation acted very indiscreetly. Messrs. Nestlé's letter to the editor of *Education* gave the impression that the initiative came from them, so it is their own fault if a misrepresentation on this point took place. As to the general principle involved, we adhere to the opinion expressed last week, though we are pleased to find that this particular firm contravened it to a much less extent than they themselves led us to suppose. Obviously, if firms of high standing start to name beds in hospitals, those of lesser repute may follow the example set; and, ultimately, since there is no hard-and-fast line of virtue in commercial affairs, incidents may follow on all-fours with the affair of Mr. Hooley's gift of plate to St. Paul's Cathedral.—ED. THE HOSPITAL.]

